#  On the Power of Additional and Complex Chromosomal Aberrations in CML

**DOI:** 10.2174/138920212802510466

**Published:** 2012-09

**Authors:** Karin M Greulich-Bode, Barbara Heinze

**Affiliations:** 1 Division Genetics of Skin Carcinogenesis, German Cancer Research Center (DKFZ), D-69120 Heidelberg, Germany; 2 University of Ulm, Institute of Human Genetics, D-89081 Ulm, Germany

**Keywords:** CML, Cytogenetics, Fish, CGH translocation, Breakpoints, Complex aberrations, Clinical impact.

## Abstract

Unregulated proliferation of mainly myeloid bone marrow cells and genetic changes in the hematopoietic stem cell system are important features in Chronic Myeloid Leukemia (CML). In clinical diagnosis of CML, classical banding techniques, fluorescence in situ hybridization (FISH) probing for the Philadelphia chromosome (Ph) or polymerase chain reaction amplifying the fusion products of the BCR-ABL fusion are state of the art techniques. Nevertheless, the genome of CML patients harbors many more cytogenetic changes. These might be hidden in subpopulations due to clonal events or involved in extremely complex aberrations. To identify these additional changes, several cytogenetic and molecular genetic techniques could be applied. Nevertheless, it has been proposed that identifying these aberrations is time consuming and costly and since they cannot be converted into a benefit for the patients, the necessity to perform these investigations has been questioned. In the times where highly specialized medicine is advancing into several areas of cancer, this attitude needs to be reassessed. Therefore, we looked at the usefulness of a combination of different techniques to unravel the genetic changes in CML patients and to identify new chromosomal aberrations, which potentially can be correlated to different stages of the disease and the strength of therapy resistance. We are convinced that the combination of these techniques could be extremely useful in unraveling even the most complex karyotypes and in dissecting different clones contributing to the disease. We propose that by doing so, this would improve CML diagnostic and prognostic findings, especially with regard to CML resistance mechanisms and new therapeutic strategies.

## INTRODUCTION

CML is a clonal myeloproliferative disorder, where an unregulated proliferation of mainly myeloid cells in the bone marrow is followed by an increase of mature granulocytes and their precursors in the blood. It is characterized by an elevated production of white blood cells, the leukocytes, especially of their uncontrolled precursor cells. These leukemic cells expand sharply in the bone marrow, displace normal hematopoesis and eventually become visible in the peripheral blood stream. As proliferation continues the white cells spill over in to the circulation. Leukemic cells in the bloodstream can cause hepatosplenomegaly, usually causing the first clinical symptoms. However, CML is often an “accidental finding” in people who do not show any symptoms. As the disease progresses, leukemia cells infiltrate the liver, the spleen, lymph nodes and other organs, thereby reducing their functionality. Additionally, normal hematopoiesis can become impaired as the disease progresses, which can result in anemia and thrombocytopenia later on, the white cell count, however, always remains high. Leukemia can be divided into acute and chronic leukemia.

 Chronic leukemia, which includes CML and CLL, is characterized by a slow progression of the disease, involving myeloid or lymphoid white leucocytes. In acute leukemia, with the myeloid (AML) or lymphoid (ALL) celltype involved, relate to the original diagnosis of leukemia and show a lot of serious medical conditions. Acute leukemia is a life threatening disease, which - if it is untreated - leads to death within a few weeks or months. Quite contrary, chronic leukemia develops mostly over several years and in the initial stadium very often is accompanied by merely mild symptoms.

 The discovery of the Philadelphia (Ph) chromosome made chronic myeloid leukemia (CML) the first cancer characterized by a nonrandom, recurrent marker chromosome [1]. This marker chromosome, which carries the BCR/ABL fusion gene, plays a major role in CML pathogenesis [2, 3] and is present in about 90% of patients with a clinical picture consistent with CML [4]. Therefore, the detection of the Ph-chromosome via polymerase chain reaction (PCR) or cytogenetic techniques confirms CML-diagnosis [5, 6].

 In general, the disease CML is divided into three phases: a chronic phase (CP), an accelerated phase (AP) and the blast crisis (BC). Typically, the CP with leucocytosis – often asymptomatic - and anemia and splenomegaly is cytogenetically characterized by the presence of a Ph chromosome. In the more advanced stages like AP (defined as 15% or more peripheral blasts and 30% or more peripheral blasts and promyelocytes [7]) and BC (defined by the presence of 30% or more blasts in the peripheral blood (PB) or bone marrow (BM)), around 80% of cells show additional secondary chromosomal aberrations [8, 9]. The disease now become acute leukemia, which can be a myeloid crisis (most common) or a lymphoid blast crisis. The best known secondary chromosomal aberrations are the double Ph chromosome, a c-Myc copy number gain due to trisomy 8, and the loss of the tumor suppressor gene p53 in the course of the formation of an isochromosome i(17q) [10, 11]. Nevertheless, additional chromosomal aberrations may accompany these known secondary aberrations and might influence the course of the disease as well. These mostly unbalanced additional secondary cytogenetic aberrations have not yet been systematically screened, but an impact with regard to occurrence in different phases has been proposed [12]. Up to now, “practically nothing is known about the mechanisms of “crossing over” or translocations” and only little is known about the additional cytogenetic changes that accompany the presence of the Ph chromosome in CML patients [12]. Therefore, it needs to be discussed, if the elucidation of the complexity of genetic changes, which can be found in CML patients, might correlate with clinical findings and the course of the disease. If so, detailed analysis would promote the field of CML diagnosis, therapy and prognosis and could thereby lead to a benefit for CML patients. Now exist guidelines to test at diagnosis and, very importantly, when to repeat tests and cytogenetics.

### Ways to Systematically Screen for Complex Aberrations Accompanying CML

 An initial step which could be taken is to screen selected blood and bone marrow samples from CML patients in different stages of the disease for hidden and complex chromosomal aberrations. Here, additionally to classical cytogenetic analysis [13] samples could be systematically investigated by fluorescence in situ hybridization (FISH, [14]), multicolor fluorescence in situ hybridization (M-FISH, [15]), classical comparative genomic hybridization (CGH [16-18] or array CGH [19]). The aim of applying the different cytogenetic methods would be to get maximum information on the karyotypic changes present in each case and at the different stages of the disease.

 Combining several cytogenetic and molecular genetic techniques has the advantage that the strengths of each of the techniques could complement the picture of the disease. (Table **1**) shows these differences which characterize the diverging techniques in regard to spatial (ability to detect even small genomic alternations) and cellular (ability to detect rare clonal events which might be important for the progression of the disease) resolution, the broadness of the view on different structural aberrations (balanced translocations as well as DNA gains and losses) and the potential to analyze the entire genome. Classical banding techniques (GTG-banding or Q-banding) are still the routine gold standard used for elucidating karyotypic abnormalities. The CGH technique has been described as the method-of-choice for detecting tumor genome imbalances, like copy number gain or losses, especially when archival, non-proliferating specimens are analyzed. Results have been reported for example for lymphomas and leukemias [20-23] and for example for myeloid leukemogenesis applying array CGH [19]. Nevertheless, only few reports describing the application of CGH in CML research have been published [24-26]. FISH using specific whole chromosome painting (WCP) or gene specific probes is by now a widely and routinely used powerful, rapid technique for analyzing karyotypic changes. One prominent example being the detection of candidate isochromosomes, e.g. i(17q) by whole chromosome paining or locus specific probes for BCR and ABL to detect the fused genes. In theory dedicated whole-chromosome painting using differentially labeled probes for several chromosomes can provide a whole-genome view of complex translocations. Nevertheless, even more complex FISH techniques like the Multicolor-FISH (M-FISH) technique, which is optimal to identify even the most complex aberrations, are used when needed for diagnostic purposes [22, 27] and already point to the usefulness of its application. By applying a combination of these techniques on routine CML cases, one would be able to detect cytogenetic changes for each and every case and thus be able to detect novel breakpoints which provide new insight into CML pathogenesis and potentially help to define new prognostic factors.

a) GTG-banding detects very complex clonal, non-clonal genetic changes in CML patients.

Classical cytogenetic analysis clearly is able to identify if patients are Ph-negative or Ph-positive. It also shows if a stable karyotype is represented in 100% of the analyzed metaphases. In more complex cases, we had been able to detect up to five different clones contributing to the cytogenetic picture of blood lymphocytes of patients. On the other hand, very complex karyotypes cannot be resolved by GTG-banding at all. Therefore, routine diagnostics already indicates that the karyotypic complexity in CML patients can vary drastically from patient to patient and that clonal aberrations can be detected as well as the presence of a Ph chromosome in the majority of the cases.

b) CGH detects blast specific genomic imbalances in CML patients.

By classical CGH analysis, gains and losses of hidden imbalances can be detected, which might be overlooked by banding techniques. CGH can, therefore, reveal additional genomic imbalances, which might be overlooked by GTG-banding. Nevertheless, CGH might fail to detect blast specific double Ph-chromosomes, because these might only be involving small parts of chromosomes. Additionally, sub-chromosomal aberrations might be visualized or overlooked by CGH depending on the percentage of total cells that certain clones have. Quite contrary, array CGH with it’s much higher resolution would allow to detect these smaller changes as well [19].

c) FISH and M-FISH experiments help to clarify ambivalent findings and unambiguously identify CML patiens’ karyotypes.

In some cases GTG-banding and CGH analysis will not be sufficient to fully unravel the karyotypic changes in CML patients. Therefore, additional FISH and M-FISH experiments can help to elucidate uncertainty in karyotypes.

### Example of a CML Patient Where Disease Progression was followed up with Different Cytogenetic Assays

 We examined the power of a systematically use of the different techniques mentioned above, each being able to detect different types of alterartions, in a patient where CML disease progression was followed up with different cytogenetic assays see Fig. (**1**). This patient was diagnosed with CML and died three years after diagnosis, after undergoing four times blast crisis of the disease. In this case, classical GTG banding detected two clones which were described as clone 1: 47,XY, +8, t(9;22)(q34;q11); Clone 2: 46,XY, t(9;22)(q34;q11) (data not shown). CGH showed a gain of 6p22-qter and 8q22-qter as well as a loss of 6p22-pter Fig. (**1A**). To further investigate the CGH findings, FISH was conducted with a whole chromosome painting (WCP) probe specific for chromosome 6 (thick arrow) and two locus specific YAC probes mapping to 6p23 (thin arrow). These FISH experiments showed three signals for chromosome 6 and only on one of these a signal for 6p23 Fig. (**1B**). Additional FISH experiments with a WCP probe specific for chromosome 8 (thick arrow)and a locus specific probe for the 8q telomere region (thin arrow)revealed two normal copies of chromosomes 8, but four signals of the telomeric region 8q. A translocation with a C-group (chromosomes 6-12) chromosome was suspected Fig. (**1C**, **1D**). M-FISH analysis confirmed the disomy of chromosome 8, additionally identified the unclear translocation as a t(6;8q) and even showed two such translocation chromosomes. Furthermore, M-FISH helped to detect a trisomy 6, two markers with a translocation t(6;8) and Ph-translocation, but failed to detect the 6p deletion Fig. (**1D**). G-banding listed a trisomy 8 next to the Ph-translocation, but no aberrant chromosome 6. The combination of all FISH results showed that the 8q22 amplification, detected by CGH, was due to the piling of two marker chromosomes t(6;8) and, therefore, “imitated” a trisomy 6, which was misinterpreted by cytogenetics as a trisomy 8. 

 Taken together, this example nicely shows how CGH, FISH and M-FISH experiments helped to clarify ambivalent findings and unambiguously identified this CML patient’s karyotype.

## DISCUSSION

 Today, chromosomal aberrations detected by classical metaphase cytogenetics, fluorescence in situ hybridization (FISH), comparative genomic hybridization (CGH, conduced in a classical way or as genomic arrays) and multicolor FISH have a great potential, besides their diagnostic value for some hematological malignancies, to be highly predictive of prognosis or responsiveness to specific therapeutics. Furthermore, invariant aberrations can be used as clonal markers to detect and follow minimal residual disease and relapse [19].

 Chronic myeloid leukemia cases are mainly characterized by the presence of the Ph-chromosome, which is characteristic for the disease [1]. Nevertheless, little is known about genetic changes, which might accompany the three phases and which may assist classification and defer from each other by the complexity and diversity of additional genetic changes. During the chronic phase (CP), G-banding easily reveals balanced translocations, whereas additional atypical unbalanced sub-chromosomal aberrations might be overlooked but picked up by CGH. During accelerated phase (AC) and blast crisis (BC) phases of the CML disease, numerical imbalances occur quite frequently. One explanation for the occurance of unbalanced aberrations during CP might be that these unbalances are not as rare as previously reported and have been underestimated in the past.

 We showed for one case of CML the benefit of applying CGH to this CML patients’ samples combined with the FISH and M-FISH method, thereby elucidating new and additional aberrations, which can be correlated with the phases of the disease. In this case, various changes in chromosome 6 were seen by CGH and M-FISH. The 6p21-p23 region might be of great importance and uniform aberrations were not found until 1989. Translocations with chromosome 6 often occur as primary aberration in AML. Nevertheless, it has been shown that a translocation t(6;9)(p23;q34) in CML is not the same as in AML and it was suggested that additional genes on 6p23 may be important for the pathogenesis of CML [28]. 

 Our example also revealed a trisomy 8, which was not detectable by CGH. Most likely, the trisomy 8 carrying clone has escaped CGH detection due to the low cell number of this clone, an effect which has been described for polyclonal tumors subjected to CGH [29]. Interestingly, trisomy 8 and other abnormalities affecting the 8q24 region are very important, because this includes the gene locus for the c-Myc gene. C-Myc is a key player in cell growth and differentiation [30] and a correlation between high c-Myc expression and CML progression has been reported [31]. It has been postulated that upregulated c-Myc expression may inhibit myeloid differentiation and may increase proliferation potential of cells [32].

 It has to be noted, that classical CGH has it’s detection limits, e.g. greater than 2 x 10^6^ basepaires (bp) for amplifications and 10-20 x 10^6^ bp for deletions [11, 17, 33]. Additionally, heterochromatin rich regions are excluded of CGH analysis due to their high copy numbers and non-specificity for distinct genomic regions [17]. However, such regions are mainly represented in the small extra Ph-chromosome 22p-q11 area, as is the same for chromosome 19, making these marker chromosomes extremely resistant to detection by CGH. Additionally, the CGH technique carries the risk to produce false positive or false negative results, according to the limits of the ratio which can be set, in our example of 1.25/0.75. This opinion differs between authors where 0.65 and 1.35 limits were used [34]. We have experienced that trends of imbalances are just as important cytogenetic data and correlate with the clinical stage of the disease. In our hands such “uncertain imbalances” represented more than 50% of the total observed aberrations in our studies. Nevertheless, CGH enabled us to find new subchromosomal aberrations. This finding is of great interest especially in view of the increasing number of published data on this subject and their prognostic implications [35]. Nevertheless, many of these drawbacks connected to classical CGH have been overcome by the array CGH technology [19].

 Combination of different cytogenetic techniques appeared to be the most effective strategy for detecting a broad range of aberrations [23, 26, 36]. Nevertheless, there is no report about the combination of these molecular-cytogenetic methods used in combination for analysis of standard diagnostic samples of CML patients [20, 36]. 

 The comparison of the methods revealed divergent results. One factor involves the different power of resolution of the methods. Each technique has its limitations and is not able to identify all aberrations. (i) GTG-banding, as a standard method, failed in some cases in terms of accuracy of diagnosis and interpretation of chromosomal abnormalities (ii) CGH was unsurpassable in detecting subchromosomal unbalanced aberrations but failed identifying the structural source of the imbalances (iii) FISH revealed to be especially sensitive in detection of structural aberrations, if the aberrant chromosome or gene to be tested was known (iv) M-FISH was strong in detecting complex rearrangements of chromosomes and in identification of marker or derivative chromosomes. However, hybridization quality and number of metaphases were and will be the most limiting factor.

 Since 2000 the benefit of combining FISH with classical cytogenetic techniques became evident [37]. Since the development of different variations of the multicolor-FISH (M-FISH) approaches, the application of even more sophisticated M-FISH techniques like multicolor banding or cenM-FISH should be taken into account [38]. We propose to also include CGH – if possible as array CGH - as this has been shown to be beneficial in CML patients [39]. Doing so in larger cohorts of CML patients would lead to very pinpointed cytogenetic diagnosis from CP to AP and impact a patient's prognosis and subsequent treatment, since therapeutic possibilities targeting the BCR-ABL translocation are constantly growing [40].

 Nevertheless, it has to be discussed that applying all four methods for each and every CML patient on a routine basis of course would be too costly and not feasible to be done in all laboratories who offer leukemia diagnosis. The great advantage of leukemia is, that here very early on study groups and leukemia networks were formed, which by now are extremely well established and have proven to be very powerful in many aspects. For example these networks serve as platforms to very quickly distribute the experiences with applying new technologies in leukemia, even including flow-karyotyping. They have set up centers where these technologies (for example M-FISH) can be offered for enrolled laboratories, so that not every laboratory needs to establish all the techniques, which very often also require extremely expensive equipment and deep knowledge. For leukemia, the design of such a study might even include the detection of microdeletions and single nucleotide changes, e.g. changes which cannot be detected by the methods mentioned above but by deep sequencing. Therefore, the feasibility to envision a larger project systematically screening CML patients in the proposed way, managed over leukemia networks is very high, probably higher than for other entities. In the times where personalized medicine is envisioned for cancers the importance of additional chromosomal aberrations in CML patients needs to be re-thought since it has the great power to lead to a much more defined diagnosis, prognosis and in the long run also to adapted therapy.

## Figures and Tables

**Fig. (1):**
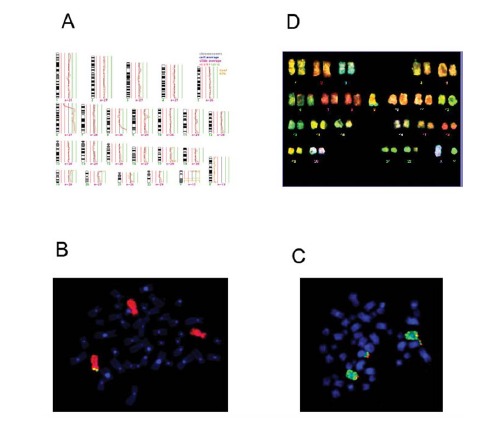
CGH, FISH, M-FISH of a severe case of CML. (**A**) CGH shows +6p22-qter and +8q22-qter as well as -6p22-pter. (**B**) FISH was done with wcp 6 (red) and YAC 6p23 (green). (**C**) FISH was done with WCP 8 (green) and telomere-8q (red). (**D**) M-FISH.

**Table 1.** Advantages and Shortcommings of Different Cytogenetic Methods

Method	Advantage	Shortcommings
Classic Banding Karyotyping (GTG Banding)	Detects Rare Clonal Events Genome Wide Detects Balanced Translocation	Labor Intensive, Automation is Costly Low Resolution Only on Fresh Material
CGH / Array CGH	Genome Wide High Resolution (Array CGH) Suitable for Automation	Rare Clonal Events Undetected Expensive (Array CGH) Balanced Translocations Undetected
M-FISH	High Resolution Suitable for Automation Detects Rare Clonal Events Identifies All Balanced and Imbalanced Aberrations throughout the Entire Genome	Costs for mFISH Probes Expensive Data Processing Resolution Depends on the Quality of Metaphases Achievable with Fresh Material
FISH with Whole Chromosomal Painting Probes (WCP) Gene / Locus Specific Probes (LSI)	WCP Genome Wide Screen is Possible Intermediate Resolution Suitable for Automation Detects Rare Clonal Events LSI High Resolution Interphase Cytogenetics Possible Does not depend on Proliferating Cells	WCP Performed as one – or two-color FISH Translocation Partners may remain unknown LSI Need to know the gene / Locus of interest
